# Silica exposures and silicosis incidence in the Western Australia mining industry

**DOI:** 10.1093/occmed/kqaf006

**Published:** 2025-07-21

**Authors:** A W Harman, P G Foley, M I Ralph

**Affiliations:** WorkSafe Mine Safety, Department of Energy, Mines, Industry Regulation and Safety, WorkSafe WA, East Perth, WA 6004, Australia; WorkSafe Mine Safety, Department of Energy, Mines, Industry Regulation and Safety, WorkSafe WA, East Perth, WA 6004, Australia; WorkSafe Mine Safety, Department of Energy, Mines, Industry Regulation and Safety, WorkSafe WA, East Perth, WA 6004, Australia

## Abstract

**Background:**

Silicosis has historically been an issue in the Western Australian mining industry.

**Aims:**

To determine the magnitude of exposures to atmospheric respirable crystalline silica (RCS) in mine workers recorded between 1986 and 2023 and if those exposures risk health effects.

**Methods:**

We used descriptive statistics to compare RCS exposures in mining job types. We identified high exposure occupations and modelled their resulting lung silica burden using known toxicokinetic parameters. These were compared with critical lung silica burdens for alveolar inflammation, soft macules, fibrosis and progressive massive fibrosis. We compared the miners’ RCS exposures with historical silicosis cases in Western Australia’s mine workers.

**Results:**

The geometric mean of more than 130 000 RCS results between 1986 and 2023 was 0.008 mg/m^3^. Exposures in exploration jobs were higher than in jobs on established mine operations (0.013 vs 0.007 mg/m^3^). Overall, exploration drilling assistant jobs and laboratory work were the two highest exposed cohorts, and modelling of steady state lung burden predicted 7.5 and 5.7 mg/lung, respectively, values an order of magnitude less than that associated with inflammation, and two orders of magnitude less than that associated with fibrosis. There have been 4 confirmed and 3 other possible cases of silicosis in more than 2 million person-years of mine work in WA since 1986.

**Conclusions:**

The low incidence of silicosis in the WA mining industry over the past 20 years is consistent with the estimated low silica lung burdens resulting from work-related exposures, which are significantly lower than the silica lung burdens typically associated with silicosis in the literature.

## Teaser

The exposure of mine workers to atmospheric respirable crystalline silica recorded in a database of over 130 000 results collected over a 38 years period was used to model the amount of silica accumulating in mine workers’ lungs. Estimates of silica in the lungs of workers with the highest silica exposures were an order of magnitude less than amounts reported as being associated with silica’s adverse effects on the human lung.

## Introduction

Dust exposure data and the incidence of silicosis amongst metalliferous miners in Western Australia (WA) has been recorded since 1925, and up to the 1970’s, there were typically 50–100 silicosis cases per year [1]. From 1925 to 1996, miners were required to have chest X-rays at regular prescribed intervals. The introduction of more stringent occupational hygiene requirements with the setting up of the WA Ventilation Board in 1974 resulted in the regulation of a workplace exposure standard (WES) for RCS of an 8 hours time-weighted average (TWA) of 0.2 mg/m^3^ throughout the WA mining industry. The oversight by the Ventilation Board resulted in a significant decline in silicosis cases in the WA mining sector, so much so that independent studies in 1993 [2], 2002 [3] and 2015 [4] found no silicosis cases in WA mine workers who had entered the workforce since 1974. The WES was reduced to 0.1 mg/m^3^ in 2005 before further reducing to the current WES of 0.05 mg/m^3^ in late 2020.

Silicosis has re-emerged in the non-mining sector in WA following the growth of the engineered stone benchtop industry. In Australia, there have been over 500 silicosis cases since 2015 in the engineered stone industry [5, 6]. WA legislation passed in 2021 now mandates health surveillance, including medical examination, spirometry, and the use of low-dose high-resolution computed tomography (LDCT) chest scan for silica health monitoring in any industry, including mining, where there is medical opinion of a risk to the worker’s health because of exposure to RCS [7]. LDCT is superior to radiographs as a diagnostic tool for detecting appearance of early indications of silicosis [8]. There is a large body of data that defines RCS exposures in WA miners but very little data on the incidence of early indications of silicosis.

The present study examines the level of RCS exposure in WA miners with the aim of identifying those mining jobs where workers may be at greater risk of developing lung pathology from RCS exposure. It models the lung burden of silica resulting from mine workers’ exposures and, using a risk assessment model, compares modelled lung burdens to that associated with pathobiological changes associated with silicosis.

**Key learning points**

**What is already known about this subject:**

- Crystalline silica dust exposure occurs in the Western Australia mining industry and was associated with a high incidence of silicosis in the early to mid-20th century, but there have been very few cases of silicosis recorded in the WA mining industry since 1974.
- Exposure to respirable crystalline silica has been systematically monitored in miners since 1986 with over
  130 000 results but has not been the subject of toxicological risk assessment.

**What this study adds:**

- Toxicokinetic modelling of silica lung burden suggests that miners’ exposures result in lung burdens at least an order of magnitude less than that associated with literature estimates for alveolar inflammation and two orders of magnitude less than those for fibrosis.
- The legislative mandating of low dose CT scans for health monitoring in 2021 has resulted in the identification of small numbers of silicosis cases and lung conditions possibly complicated by silica.

**What impact this may have on practice or policy:**

- Health monitoring should focus on mine workers with a long work history in high exposure jobs, those being workers most at risk of silicosis.
- Where LDCT identifies early signs of silicotic disease, intervention to either reduce or eliminate further exposure would be warranted in order to either halt or reverse the course of disease.
- Workforce awareness on the hazards of silica dust, industry compliance with exposure standards and enforcement activities by the Government Regulator have been effective in virtually eliminating silicosis in the WA mining industry.

## Methods

Since the mid-1970’s, WA mine safety legislation has required mine operators to sample worker exposures to a range of atmospheric contaminants and report results to the mine safety regulatory authority, presently the Department of Energy, Mines, Industry Regulation and Safety (DEMIRS). DEMIRS maintains a Safety Regulation System database (SRS Database), into which mine operators submit hygiene sampling data, including atmospheric contaminants and biological monitoring [9]. DEMIRS monitors these inputs for compliance and data integrity.

Personal results of RCS samples from the breathing zone have been recorded since 1986 including details such as date, company, worker, job type and location in mine [1]. Information on the mined commodities is in Supplementary Table S1 (available as Supplementary data at *Occupational Medicine* online). Personal respirable dust samples are collected per Australian Standard (AS 2985), using ISO 7708 compliant samplers and trained technicians. The RCS content is analysed by National Association of Testing Authorities accredited laboratories using X-ray diffraction or infra-red spectroscopy. By the end of 2023, more than 134 000 personal RCS results have been recorded in the SRS Database.

Descriptive statistics were applied to personal monitoring results of exposure measured as the average concentration of RCS over the sampling period where the sampling period is at least 4 hours, preferably at least half the shift and ideally near the full shift length. Compliance with the statutory WES was measured by per cent of results exceeding the WES. Both exposure and compliance statistics were examined for year, mine type, commodity mined, job type and location in a mine. Statistical parameters calculated were median, geometric mean (GM), and geometric standard deviation (GSD), the upper 95% reference range of the GM (GM × GSD^2^) and the non-parametric upper 95% tolerance limit of a distribution. Twenty results exceeding 10 mg/m^3^ were excluded from statistical analysis as unreliable/contaminated. The unpaired *t*-test was used for comparisons of two groups. One-way analysis of variance on log_e_-transformed RCS data was used to determine if any group differed significantly from the overall group mean and post hoc differences between group members determined using the Scheffé method. Statistical analyses and graphics were conducted using Microsoft Excel for Microsoft 365 MSO (Ver. 2308).

There is a theoretical relationship between exposure to the agent and the amount retained in the body when the rate of absorption balances the rate of elimination [10]. The exposure concentration of RCS was used to estimate the amount of silica in the lungs when the amount of RCS being deposited balances the amount of silica excreted (steady state). Modelling of the amount of silica residing in lungs at steady state, resulting from exposure to a specified concentration of RCS, is calculated based on known physiological and toxicokinetic parameters in humans estimated by others [11, 12]. The formula for the total silica deposited into the alveolar region up to steady state is as follows: ‘Lung silica load at steady state = [exposure] × [fraction of full year at workplace] × [rate of air intake at work] × [exposure duration at work] × [time to steady state] × [fraction of inhaled silica retained in alveolar region]’.

The parameters were calculated as follows: ‘exposure’ is the concentration of RCS (mg/m^3^); ‘fraction of full year at workplace’ is 0.31 based on working 2688 hours (12 hours shift, 2 weeks on/1 week off for 48 weeks) out of 8760 hours in the full year; ‘rate of air intake at work’ = 0.019 m^3^/min [13]; ‘exposure duration at work’ = constant exposure at specified RCS concentration for full 12 hours shift; ‘time to steady state’ = 2310 days [12]; and ‘fraction of RCS inhaled and retained in alveolar region’ = 0.133 [12].

It is assumed that the time to reach steady state is approximately five half-lives if the chemical exposure is at regular intervals, no matter the number of exposures, the exposure size or the exposure interval [10]. The half-life of RCS in humans has been estimated as 462 days based on an elimination rate constant of 0.0015 per day [12].

An estimate of the lung burden at steady state with regular exposures to a known concentration of RCS was calculated assuming a one-compartment model [14]


$$\begin{array}{l} Lung\;burden\;at\;steady\;state=\frac{F\;x\;A}{ke\;x\;T} \end{array}$$

where *F* is the fraction of inhaled silica reaching the alveoli; *A* is the amount of RCS inhaled in one shift, equal to [exposure concentration] × [rate of air intake at work] × [shift length in minutes]; *ke* is the elimination rate constant; *T* is the dose interval (3.2 days, being the inverse of ‘fraction of full year at workplace’).

In the context of lung load, reference to lung includes the total amount in all lung lobes.

Cumulative exposure (concentration × time) is a commonly used measure of risk. The SRS Database does not record the length of time an individual has worked in mining, so there is no measurement parameter of time to which any individual worker or group of workers have been exposed to RCS. An alternative measure of exposure is the amount of silica in the lungs. An approach used in the present study is to estimate of the ‘amount of silica per human lung’ as the measure of risk using methodology adapted largely from research work at the Institute of Occupational Medicine by CL Tran and colleagues. Using a combination of animal studies and human autopsy data, they estimated a silica load in the human lungs of 200–1000 mg is associated with minimal cellular response and hence minimal inflammation [11, 15]. The critical lung burden for inflammation and for fibrosis has been calculated using a biologically based mathematical model of retention and clearance of silica in the rat lung [15]. The threshold lung burden for inflammation in humans was estimated from that data as 150 mg/lung, and 1300 mg/lung for fibrosis.

Silica lung loads between 700 and 1700 mg have been estimated to be associated with soft macules in coal miners [16]. Soft macules develop at exposure concentrations high enough to overload alveolar clearance mechanisms but do not necessarily progress to more severe disease. They have not been associated with pulmonary symptoms or to significant changes in lung function, such as in FEV_1_ [17]. Lung load associated with fibrosis and progressive massive fibrosis in former coal miners is estimated to be 4500 and 4800 mg, respectively [16]. The working lifetime quartz exposures proposed as the minimum critical silica lung burden associated with reduced pulmonary clearance and increased neutrophilic inflammation in humans have been predicted as those with a mean of 390 mg/lung [18].

The lower estimates of the above referenced animal studies and human autopsy studies in UK coal miners suggest that conservative values for the critical lung loads of silica in human lung are: inflammation 150 mg [15]; soft macules 700 mg [16]; fibrosis 1300 mg [15] and progressive massive fibrosis 4800 mg [16].

## Results

**Figure 1.**
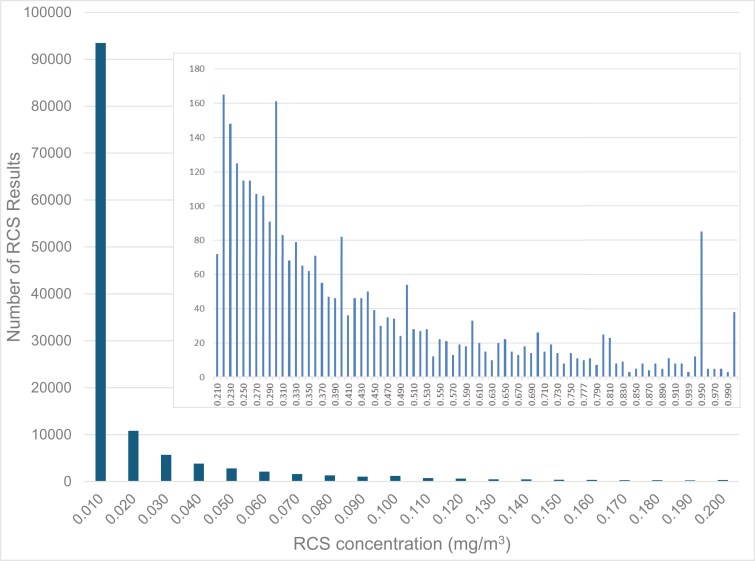
Frequency distribution of RCS concentration results in WA mining workers for the period 1986–2023 (*n* = 131 454). Insert shows the upper range of results 0.21–1.0 mg/m^3^. *X*-axis values represent the concentration range including the indicated value. There were 93 486 results (72%) less than or equal to 0.01 mg/m^3^.

**Figure 2.**
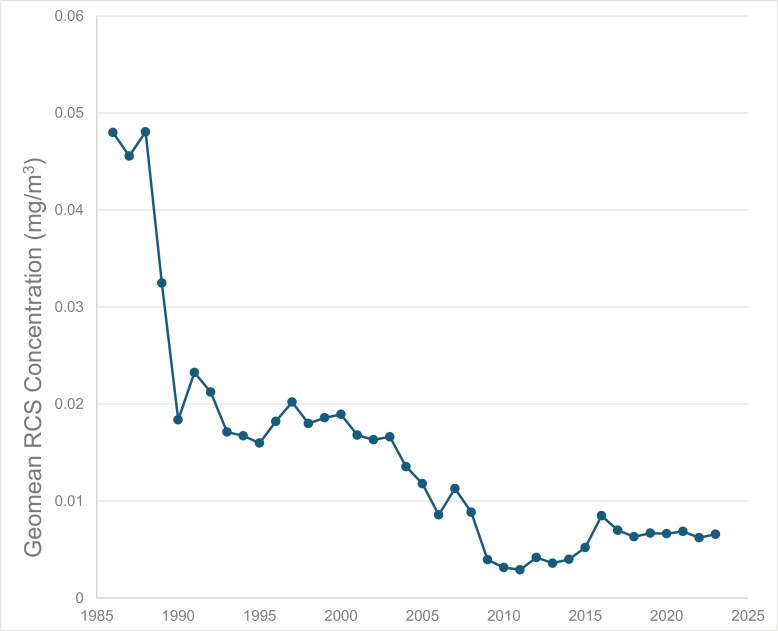
Annual GM of RCS results in mine workers’ personal samples for years 1986–2023 in WA.

The overall distribution of RCS results for the entire data set (1986–2023) is heavily skewed and significantly different from a normal distribution (Jacque–Bera normality test, *P* < 0.001) with the vast majority at or below 0.01 mg/m^3^ (Figure 1). A log_e_-probability plot confirmed that the data distribution is close to log_e_-normal (*P* = 0.023). There is a clear downward trend in GM for RCS on an annual basis in the period 1986–2023 (Figure 2). Since 2008, the GM for RCS on an annual basis has been below 0.01 mg/m^3^.

Analysis of the RCS data in the SRS Database since 1986 demonstrates that the WA mining industry has substantially complied with the applicable WES (Supplementary Table S2, available as Supplementary data at *Occupational Medicine* online). The WES applied in the SRS Database was initially an 8 hours TWA of 0.1 mg/m^3^ being a duration of 8 hours a day/40 hours week and was reduced to an ES of 0.05 mg/m^3^ in late 2020. The most common shift pattern is 12 hours per day, 2 weeks on/1 week off. In such circumstances, the 8 hours TWA is adjusted. Therefore, for example the WES of 0.05 mg/m^3^ is adjusted to an adjusted workplace exposure standard (AES) of 0.036 mg/m^3^ to account for the longer shift. Since 1986, exceedances of the AES have averaged 4% for RCS. A change of the WES to 0.05 mg/m^3^ has resulted in 5% of results exceeding the AES in the period 2021–2023. For all RCS results 1986–2023, 90% are at or below 0.05 mg/m^3^. For years 2021–2023, the corresponding value is 96% (Supplementary Table S3, available as Supplementary data at *Occupational Medicine* online). Where exceedances have occurred, their magnitude has not been excessive, being typically in the range 150–200% of the AES. That is, while there have been exceedances, they are relatively few and relatively small.

Work areas within mining are grouped into Location Codes in the SRS Database. RCS results under each Location Code are summarized in Table 1. One-way analysis of variance (ANOVA) demonstrates statistically significant differences between the GMs of the work area location categories. Post hoc comparisons identify the highest exposure Location as surface exploration, followed by underground mining, surface mining, ore treatment plants and open pit activities (Table 1). The SRS Database can also be examined by occupation/job type. Jobs in underground mining had the highest GM, followed by those involving ore treatment and surface mining operations (Table 2). The range of concentrations in the three highest occupation codes was 0.006–0.020 mg/m^3^, corresponding to estimated silica burdens at steady state of 2.1–7.5 mg/lung (Table 3).

**Table 1.** Geometric mean, geometric standard deviation and upper 95% reference range for RCS concentration in 10 Location Codes

	Location	*N*	GM of RCS^#^ (mg/m^3^)	Geometric standard deviation of RCS	Upper 95% reference range (mg/m^3^)
1.	Surface exploration	2807	0.013	5.095	0.345
2.	Underground mining	16 682	0.009	3.208	0.089
3.	Surface mining general	12 924	0.007	3.849	0.106
4.	Treatment Plant	22 945	0.007	3.632	0.088
5.	Open Pit	23 519	0.007	3.500	0.081
6.	Workshop surface mining	8108	0.004	3.129	0.048
7.	Railways	322	0.005	3.487	0.060
8.	Power Plant	365	0.005	2.814	0.035
9.	Crushed ore areas	4009	0.004	3.332	0.049
10	Administration	3164	0.004	2.824	0.032

^#^ANOVA of log_e_ transformed data, *F* = 343, *F*_crit_ = 2.407, *P* < 0.001 for Location. Location comparisons show significant differences (*P* < 0.001) in sequential Scheffé test post hoc comparisons of GMs as between 1 v 2, 2 v 3, 3 v 4, 5 v 6, and 9 v 10. Data from SRS database recording Location of submitted RCS results (2001–2023).

**Table 2.** Geometric mean, geometric standard deviation and upper 95% reference range for RCS concentration in 9 Occupation Codes. Data from results in the SRS Database for the period 1986–2023

Occupation code	GM of RCS^a^ (mg/m^3^)	Geometric standard deviation	Upper 95% reference range (mg/m^3^)
Underground mining production	0.011^b^	4.049	0.179
Ore treatment	0.010^b^	5.281	0.281
Surface mining production	0.009^b^	4.993	0.229
Metal working	0.006	3.896	0.089
Management/Supervisory	0.006	3.488	0.070
Railway	0.005	5.756	0.168
Electrical	0.005	3.388	0.059
Miscellaneous trades	0.005	3.296	0.056
Stores and Warehouse	0.005	3.309	0.051

^a^ANOVA for GM, log_e_ transformed data, *F* = 520, *F*_crit_ = 1.94, *P* < 0.001.

^b^Significantly greater than GMs for non-asterisk Occupation Codes, Scheffé test, *P* < 0.001.

**Table 3.** Estimates of total lung load of silica at steady state based on the geometric mean and upper 95% reference range (a) total deposited alveolar silica and (b) lung load with an elimination rate constant of 0.0015 per day

Reference values: pathology associated with lung burden of silica in humans						Lung silica (mg/lung)	
Inflammation						150	
Soft macules						700	
Fibrosis						1300	
Progressive massive fibrosis						4800	
RCS concentration and lung load	Number of results	GM of RCS conc. (mg/m^3^)	(a) Silica deposition at steady state (mg/lung)	(b) GM of lung load at steady-state (mg/lung)	Upper 95% reference range (mg/m^3^)	(a) Silica deposition at steady state—upper 95% ref range (mg/lung)	(b) Lung load at steady-state—upper 95% ref range (mg/lung)
**All RCS data**							
RCS data 1986–2020	115 238	0.009	12	3.4	0.22	288	83
RCS data 2021–2023	16 965	0.006	8	2.3	0.04	52	15
**Surface exploration** **(2000** ^a^–**2023)**							
Drillers	554	0.014	18	5.3	0.38	495	143
Drillers assistant	986	0.020	26	7.5	0.65	847	244
All other jobs	1002	0.008	10	3.0	0.11	144	41
**Underground mining production (1986**–**2023)**							
Underground miners	4799	0.013	16	4.7	0.22	282	46
Loading/transport underground	9701	0.012	15	4.4	0.20	265	42
Long hole drillers (underground)	3450	0.010	12	3.6	0.13	167	27
Ground/roof support (underground)	817	0.009	12	3.4	0.15	200	31
Services (underground)	2642	0.009	12	3.5	0.13	165	27
Diamond driller/raiseborers	1305	0.008	10	2.9	0.10	126	21
**Surface mining production (1986**–**2023)**							
Sampling, assay, laboratory	9311	0.015	20	5.7	0.66	854	138
Processing ore	19 930	0.009	12	3.6	0.23	298	48
Final product/transport	2495	0.006	8	2.3	0.09	117	19
Mobile plant	2369	0.006	7	2.1	0.08	194	17

^a^date surface exploration first introduced as a parameter in the SRS Database.

The SRS Database introduced a stand-alone category for the ‘exploration’ parameter in 2000. This parameter refers to all surface mining activity that is on a lease, but not on an operating mine site. The GM for RCS concentration is significantly higher in exploration work compared with work on operating mining sites (Table 4a). Consistent with the higher exposure in exploration work, exceedances of the WES were 4-fold higher in exploration compared with those in mining-only (Table 4a). Within the cohort of surface exploration workers, those engaged in drilling jobs had higher exposures to RCS when compared with other job types engaged in exploration work. Driller’s assistant jobs in exploration were the highest exposure group with an estimated silica burden of 7.5 mg/lung (Table 3). The driller and driller’s assistant job types in exploration were associated with a more than 3-fold higher exposure to RCS at the upper end of the 95% distribution compared with those in other exploration jobs (Table 4b).

**Table 4.** Comparisons of compliance and RCS concentration in exploration job types with mining only job types (a), and comparison between job types within exploration (b), for the period 2000–2023

RCS Data	*N*	GM of RCS (mg/m^3^)	Upper 95% reference range of RCS distribution (mg/m^3^)	Upper 95% tolerance limit of RCS distribution (mg/m^3^)	% results exceeding AES
**(a) Mining v exploration**					
Mining only	93 715	0.007	0.083	0.060	3%
Exploration	2542	0.013^a^	0.329	0.260	12%
**(b) Exploration job sub-groups** ^b^					
Driller’s assistants	986	0.020	0.650	0.410	58%
Drillers	554	0.014	0.380	0.350	15%
All others	1002	0.008	0.111	0.080	5%

^a^Significantly different from mining only, *t*-test (two tailed), *t* = 19.01, *P* < 0.001.

^b^ANOVA, *F* = 87.1, *F*_crit_ = 3.0, *P* < 0.001; Scheffé test comparing GMs: Drillers Assistants > Drillers > Other jobs (*P* < 0.001).

Following a request by the WorkSafe WA Commissioner, information on the prevalence of silicosis in WA mine workers was provided by the workers compensation statutory authority, WorkCover WA. Since the introduction of LDCT scans for health surveillance in 2021, there have been 7 mine workers identified with evidence of silica in the lungs. Four cases had signs of the early lung changes consistent with silicosis. In the other 3, silicosis was less clear due to the possibility of other concomitant lung diseases. This is in the context of the WA mining workforce accounting for more than 2 million person-years since 1986 (data not shown).

## Discussion

The data modelling of RCS exposures in WA mine workers indicates that lung silica burden is much lower than levels linked to lung disease, aligning with previous findings of an absence of silicosis in the WA mining industry [2–4] and the low incidence in the more recent LDCT chest screening.

The present study shows a significant decrease in RCS exposure between 1986 and the mid-2000s, with exposure levels stabilizing over the last 2 decades. This decline is likely due to increased focus on health issues, improved mining practices and better dust control. Since the mid-1990s, RCS exposure has largely been below the current WES for RCS of 0.05 mg/m^3^ (Figure 2) and the applicable AES. However, certain roles, such as drillers and laboratory workers, show higher rates of exposure above AES, especially in exploration jobs (Table 1).

The pathobiology leading to silicosis of the lung is a complex process in which the respired silica particles are ingested by alveolar macrophages and their subsequent triggering of inflammation, generation of nodular lesions and irreversible fibrosis [19]. Autopsy studies in former coal miners and the extrapolation of the animal studies to humans have been used to correlate total lung loads associated with the stages of silicosis from inflammation, formation of soft macules, fibrotic nodules and progressive massive fibrosis in human lung [11, 12, 15–18].

The highest exposures by job type are shown in Table 3 and include sampling, assaying and laboratory work, exploration drilling, underground drilling and excavation work. These jobs all involve dusty work, and perhaps not unexpectedly, a high level of inhalable dust is a rough indicator of likely high RCS exposures. Indeed, there is a significant correlation between inhalable dust exposure and RCS exposure within the job types shown in Table 2 (Supplementary Figure S1, available as Supplementary data at *Occupational Medicine* online). Exploration work can be particularly dusty, takes place in remote locations and possibly not subject to the supervisory rigour that occurs on mine sites. The lung loads at the GM for these job types range from 2.1 to 7.5 mg/lung (Table 3), with drilling jobs and laboratory jobs being identified as obvious targets for future intervention measures to reduce further exposure. The highest two exposure job types (exploration drillers and laboratory workers) had modelled estimates of lung load GMs of 7.5 and 5.7 mg/lung, respectively, which are at least an order of magnitude less than that associated with inflammation and two orders of magnitude less than that associated with fibrosis.

The model in this study is conservative, assuming continuous RCS exposure at the GM concentration for an entire shift, for at least 5 half-lives without accounting for factors such as service length, breaks, holidays, training or proper PPE use, all of which could reduce overall exposure. The RCS elimination rate is not expected to be affected by the exposure concentrations in this study as they are well below those known to impair silica elimination [18]. RCS exposures associated with dry cutting engineered stone have been recorded with average concentrations of about 1 [20] and 44.6 mg/m^3^ over 30 minutes [21]. At these exposures, the model in the present study would suggest lung loads two orders of magnitude higher than those in Table 3 and sufficient to be associated with lung pathology. The present study confirms the very low risk for mine workers contracting silicosis in the WA mining workforce based on the measured exposure to RCS, the resulting modelled lung burden of silica, the literature estimates of silica lung burden associated with silicosis and the small number of workers’ compensation cases for silicosis from a workforce of over 2.1 million person-years since 1986. It is consistent with two epidemiological studies in 2002 and 2017 by De Klerk et al. that found no cases of silicosis in the WA mining industry [3, 4].

Using the GM as a risk measure has limitations. While Table 3 shows that the upper 95% reference range of modelled silica lung burdens is generally below the threshold for chronic alveolar inflammation, certain workers may face consistently higher RCS exposure due to unique local factors such as mining environment, dust control, ventilation, worker activity and local conditions. Therefore, the model may underestimate risk for workers with higher exposure levels or those more susceptible due to genetic factors, pre-existing lung conditions or other health issues. Therefore, the present results reinforce the continued need for regulatory compliance and controls. Monitoring is warranted in the form of hygiene improvements to reduce RCS exposure in high-risk groups such as exploration drilling and laboratory assay workers in combination with the use of medical surveillance and chest LDCT to identify early stages of disease. While there is no treatment currently available to reverse fibrosis, such monitoring should allow intervention to prevent progression of the early stages of the silicosis or even possibly the reversal of the disease process [22].

## Supplementary Material

kqaf006_suppl_Supplementary_Tables_S1-S2_Figures_S1
