# Supplementary material for: Silica exposures and silicosis incidence in the Western Australia mining industry
Source: Occup Med (Lond). 2025 Jul 21;75(5):227–34. doi: 10.1093/occmed/kqaf006 (PMC12370399; doi:10.1093/occmed/kqaf006)
Supplement: kqaf006_suppl_Supplementary_Tables_S1-S2_Figures_S1 [file kqaf006_suppl_supplementary_tables_s1-s2_figures_s1.docx]

Supplementary materials

### Silica exposures and silicosis incidence in the Western Australia mining industry

Supplementary Table S1

Supplementary Table S2

Supplementary Table S3

Supplementary Figure S1

| Table S1. Number of results (and percentage of total results) recorded in the SRS Database for respirable crystalline silica (RCS) for years 1986-2023 by mining commodity type.   \| **Commodity** \| **No. of RCS results** \| **% of total results** \| \| --- \| --- \| --- \| \| Gold \| 57209 \| 43.4 \| \| Iron Ore \| 33565 \| 25.5 \| \| Nickel \| 11680 \| 8.9 \| \| Construction Materials \| 6166 \| 4.7 \| \| Copper - Lead - Zinc \| 4444 \| 3.4 \| \| Tin - Tantalum - Lithium \| 4031 \| 3.1 \| \| Diamond \| 3197 \| 2.4 \| \| Bauxite - Alumina \| 3072 \| 2.3 \| \| Heavy Mineral Sands \| 2592 \| 2.0 \| \| Silica - Silica Sand \| 1922 \| 1.5 \| \| Coal \| 1001 \| 0.8 \| \| Manganese Ore \| 783 \| 0.6 \| \| Phosphate \| 388 \| 0.3 \| \| Talc \| 282 \| 0.2 \| \| Rare Earths \| 247 \| 0.2 \| \| Limestone - Limesand \| 207 \| 0.2 \| \| Clays \| 201 \| 0.2 \| \| Salt \| 198 \| 0.2 \| \| Chromite - Platinoids \| 120 \| 0.1 \| \| Dimension Stone \| 108 \| 0.1 \| \| Vanadium - Titanium \| 101 \| 0.1 \| \| Gypsum \| 77 \| 0.1 \| \| Chemicals \| 69 \| 0.1 \| \| Tungsten - Molybdenum \| 41 \| <0.1 \| \| Diatomite - Spongolite \| 36 \| <0.1 \| \| Pigments \| 23 \| <0.1 \| \| Silver \| 17 \| <0.1 \| |
| --- | --- | --- | --- | --- | --- | --- | --- | --- | --- | --- | --- | --- | --- | --- | --- | --- | --- | --- | --- | --- | --- | --- | --- | --- | --- | --- | --- | --- | --- | --- | --- | --- | --- | --- | --- | --- | --- | --- | --- | --- | --- | --- | --- | --- | --- | --- | --- | --- | --- | --- | --- | --- | --- | --- | --- | --- | --- | --- | --- | --- | --- | --- | --- | --- | --- | --- | --- | --- | --- | --- | --- | --- | --- | --- | --- | --- | --- | --- | --- | --- | --- | --- | --- | --- |
|  |

Supplementary Table S2. Compliance with adjusted workplace exposure standard* (AES) of the period , median and geometric mean (GM) for the time-weighted average of respirable crystalline silica (RCS) for periods between 1986-2023

| **Period** | **RCS exposure standard**  **(mg/m^3^)** | **No. of RCS results** | **No. of exceedances of AES** | **Compliance**  **(% results exceeding AES)** | **Median RCS**  **(mg/m^3^)** | **Geometric mean RCS**  **(mg/m^3^)** |
| --- | --- | --- | --- | --- | --- | --- |
| 1986-2015 | 0.1 | 87101 | 4343 | 5 | 0.010 | 0.009 |
| 2016-2020 | 0.1 | 28137 | 619 | 2 | 0.007 | 0.007 |
| 2021-2023 | 0.05 | 20025 | 1098 | 5 | 0.005 | 0.006 |
| 1986-2023 | mixed | 135263 | 6060 | 4 | 0.010 | 0.008 |

*exposure standard adjusted for shift length and shift pattern on an individual basis for each worker’s RCS result using the Québec Model

Supplementary Table S3. RCS results by concentration range

| Years 1986-2023 |  |  |  |
| --- | --- | --- | --- |
| **RCS (mg/m^3^)** | **No. of results** | **% of total results** | **Cumulative % of results** |
| >10 | 18 | 0.02 | 0.02 |
| >1-10 | 415 | 0.3 | 0.32 |
| >0.5-1.0 | 706 | 0.5 | 0.82 |
| >0.1-0.5 | 6508 | 5.0 | 5.82 |
| >0.05-0.1 | 7225 | 5.5 | 11.32 |
| >0.02-0.05 | 12285 | 9.4 | 20.7 |
| =<0.02 | 103750 | 79.3 | 100 |
| Years 2021-2023 |  |  |  |
| **RCS (mg/m3)** | **No. of results** | **% of total results** | **Cumulative % of results** |
| >10 | 2 | 0.01 | 0.01 |
| >1-10 | 2 | 0.01 | 0.02 |
| >0.5-1.0 | 17 | 0.11 | 0.13 |
| >0.1-0.5 | 213 | 1.34 | 1.47 |
| >0.05-0.1 | 357 | 2.25 | 3.72 |
| >0.02-0.05 | 1138 | 7.16 | 10.88 |
| =<0.02 | 14172 | 89.12 | 100 |

Supplementary Figure S1. Relationship between geometric means of inhalable dust exposure and RCS exposure of the 9 job types shown in Table 2 of main paper.

Data from 1991 (date inhalable dust first introduced as a parameter in SRS Database) to 2023.

Regression analysis, t=2.67, P<0.0.05. Correlation analysis, Pearson r = 0.78, P<0.001
